# 

**Published:** 1909-06-26

**Authors:** 


### BOOKS RECEIVED.

THE SCIENTIFIC PRESS.

"Mrs. Blossom on Babies." By Helen S. Cooper Hodgson, A.R.San.I.

"Burdett's Hospitals and Charities." By Sir Henry Burdett, K.C.B., K.C.V.O.

J. B. LIPPINCOTT CO.

"Essentials of Medicines." By Charles P. Emerson, M.D.

"International Clinics." Vol. I. Nineteenth series. Edited by W. T. Longcope, M.D., Philadelphia, U.S.A.

JOHN WRIGHT AND SONS, LTD.

"Golden Rules of Anæsthesia." Series No. 14. By R. J. Probyn-Williams, M.D.

FUNK AND WAGNALLS CO.

"Parsimony in Nutrition." By Sir James Crichton-Browne, M.D., LL.D., F.R.S.

HENRY FROWDE AND HODDER AND STOUGHTON.

"Sprains and Allied Injuries of Joints." By R. H. Anglin Whitelocke, M.D., M.C., F.R.C.S.

"Medical Inspection of Schools." By A. H. Hogarth, M.B., etc.

"Rose of the Wilderness." By S. R. Crockett.

"Aseptic Surgery," by Charles B. Lockwood, F.R.C.S.

### THE GRADUATES' MEDICAL SCHOOLS.

DIARY FOR THE WEEK, JUNE 28 to JULY 3.

### ROYAL SOCIETY OF MEDICINE, 20 Hanover Square, W.

**At 8 p.m.**

**June 28, Odontological Section.** Paper, Mr. Percy Pickerill: "Radicular Aberrations."

**Casual Communications.**

Mr. J. T. Carter, "A Note on the Ameloblast Cells in Esox,"

Mr. W. James and Mr. J. G. Forbes, "An Epithelial Odontome."

Mr. J. L. Payne, "An Interdental Splint."

### LONDON SCHOOL OF CLINICAL MEDICINE, Seamen's Hospital, Greenwich, S.E.

**At 3.15 p.m.**

**June 28,** Mr. Wm. Turner, **The After-treatment of Cases of Abdominal Section.**

**At 2.15 p.m.**

**June 29,** Dr. R. Wells, **Points in the Diagnosis and Treatment of Aortic Disease.**

### NATIONAL HOSPITAL FOR THE PARALYSED AND EPILEPTIC, Queen Sq., Bloomsbury, W.C.

**At 3.30 p.m.**

**June 29,** Mr. Armour, **Surgery of the Nervous System.**

**July 2,** Dr. Jas. Taylor, **Clinical Lecture on the Spinal Cord.**

### NORTH-EAST LONDON POST-GRADUATE COLLEGE, Prince of Wales's General Hospital, Tottenham, N.

**At 4.30 p.m.**

**June 29,** Dr. R. M. Leslie, **The Influence of Sex in Disease.**

### THE HOSPITAL FOR SICK CHILDREN, Great Ormond Street, W.C.

**At 4 p.m.**

**July I,** Dr. Hutchison, **Colitis in Childhood.**

### CENTRAL LONDON THROAT AND EAR HOSPITAL, Gray's Inn Road, W.C.

**At 3.45 p.m.**

**June 29,** Dr. Andrew Wylie, **The Larynx.**

### THE POST-GRADUATE COLLEGE, West London Hospital, Hammersmith, W.

**At 10 a.m.**

**June 28 and July I,** Surgical Registrar, **Demonstration.**

**July 2,** Medical Registrar, **Demonstration.**

**At 12 noon.**

**June 28,** Dr. Bernstein, **Pathological Demonstration.**

**At 12.15 p.m.**

**June 29,** Dr. Pritchard, **Practical Medicine.**

**June 30 and July 3,** Dr. Grainger Stewart, **Practical Medicine.**

**At 5 p.m.**

**June 28,** Mr. Pardoe, **Diagnosis of Surgical Diseases of the Urinary System.**

**June 29,** Mr. Etherington-Smith, **Clinical Lecture.**

**June 30,** Dr. Low, **Bilharziosis.**

**July I,** Mr. Dunn, **Cases of Eye Diseases.**

**July 2,** Dr. Seymour Taylor, **Valvular Disease: Aortic Regurgitation.**

### MEDICAL GRADUATES' COLLEGE AND POLYCLINIC, 22 Chenies Street, W.C.

**At 5.15 p.m.**

**June 28,** Dr. F. J. McCann, **Irritable Bladder.**

**June 29,** Mr. T. H. Openshaw, **The Treatment of Lateral Curvature.**

**June 30,** Dr. C. Riviere, **The Diagnostic and Therapeutic Use of Tuberculin.**

**July I,** Dr. Jas. Collier, **Aphasia according to the New Doctrine.**

**At 4 p.m.**

**June 28,** Dr. J. H. Sequeira, **Skin.**

**June 29,** Dr. Essex Wynter, **Medical.**

**June 30,** Mr. Mower White, **Surgical.**

**July I,** Sir Jonathan Hutchinson, **Surgical.**

**July 2,** Mr. Hunter Tod, **Ear, Nose, and Throat.**

THE HOSPITAL June 26, 1909.

*Name* ..........................................

*Address* ..........................................

..........................................

This Coupon must accompany manuscript or contributions intended for The Hospital.

